# Clinicopathological study of 9 cases of prostate cancer involving the rectal wall

**DOI:** 10.1186/s13000-017-0599-2

**Published:** 2017-01-17

**Authors:** Tao Tang, Zhengduo Yang, Dan Zhang, Jie Qu, Guang Liu, Shiwu Zhang

**Affiliations:** Department of Pathology, Tianjin Union Medical Center, Jieyuan Road, Hongqiao District, Tianjin, 300121 People’s Republic of China

## Abstract

**Background:**

Prostate cancer involving the rectal wall is rare and may lead to diagnostic pitfalls.

**Case presentation:**

Out of 9504 patients with rectal tumors between January 2003 and January 2015, 9 patients (elderly with a mean age of 74 years) with prostate cancer involving the rectal wall were clinically misdiagnosed with rectal cancer. The lesions were located in the rectum, and included 3 circumferential rectal masses, 1 ulceration lesion, 1 crater-like mass, and 4 protruding lesions. Specimens were acquired using biopsy, fine needle aspiration, or resection. The initial symptoms of these patients included rectal urgency, bowel obstruction, and lower gastrointestinal bleeding. Prostate-related symptoms were not obvious. Histologically, 2 cases showed cancer cell invasion in the mucosa, 1 showed transmural invasion from the mucosa to subserosal soft tissues, and 7 cases had submucosa and muscularis propria involvement. All the 9 cases had muscularis propria involvement. However, there were no intraepithelial neoplasias in the mucosal layer, which is reminiscent of rectal carcinoma. The tumors consisted of small-sized or foamy cells that formed acinus-like, duct-like, and cribriform-like structures. We conducted histological staining and an immunohistochemical analysis for CDX-2, prostate-specific antigen (PSA), P504s, villin, carcinoembryonic antigen, CK-pan, cytokeratin 20, and Ki-67. All tumors were PSA and CK-pan positive, 5 of 9 tumors were P504s-positive, and all tumors were negative for the other markers. All patients underwent standard therapy for prostate cancer after the definitive pathological diagnosis. As of March 31, 2015, 8 patients were alive and 1 had died of prostate cancer 6 months posttreatment.

**Conclusions:**

Adenocarcinoma appearing in the rectal wall is not always rectal carcinoma. It is necessary to perform a differential diagnosis for prostate cancer in cases of rectal malignant tumors in elderly male patients. Any treatment should be postponed until the final definitive diagnosis is reached.

## Background

Prostate cancer is the second most frequently diagnosed cancer and the fifth leading cause of cancer death among men worldwide [1]. In China, the incidence rate of prostate cancer was 1.6/100 000 individuals. However, its incidence has been increasing each year [2]. Prostate cancer risk factors include a family history of the disease, ethnicity, and in particular older age [3], with most cases occurring in men older than 50 years [4–6]. With more comprehensive screening techniques being increasingly used in China, the incidence of prostate cancer may rapidly increase in the future [2].

The prostate is located in the pelvis, under the urinary bladder and in front of the rectum. Because of its location, prostate cancer often affects urination, ejaculation, and more rarely, defecation. Prostate cancer may invade the nearby organs including the rectum, bladder, and ureters, and metastasize to the bones and lymph nodes [7–9]. The presenting symptoms include difficulty urinating, blood in the urine, and pelvic pain [10, 11]. Because of its proximity to the rectum, prostate cancer can be misdiagnosed as rectal cancer.

There is a thick capsule (Denonvilliers’ fascia) between the prostate and rectal wall [12], and prostate cancer accompanied by rectal invasion is rare [13, 14]. In the present study, we retrospectively analyzed 9504 cases diagnosed as rectal cancer in our hospital from 2003 to 2015, and report the clinicopathological characteristics of 9 cases of prostate cancer with rectal wall invasion misdiagnosed as rectal cancer. In these 9 patients, the initial symptoms in 8 patients were rectal urgency, bowel obstruction, and lower gastrointestinal bleeding, and prostate-related symptoms were not obvious. A definitive diagnosis of prostate cancer invading the rectum can be made based on the patients’ history, the morphological features of the cancer, and immunohistochemical (IHC) analyses. Furthermore, the serum prostate-specific antigen (PSA) levels and the ratio of free PSA to unbound PSA can be helpful in avoiding a clinical misdiagnosis [15].

## Case presentation

### Patients

This study was approved by the Institutional Review Board of Tianjin Union Medicine Center, and the patients’ anonymity has been maintained. The surgical pathology database at the Department of Pathology (2003–2015) was searched for cases of prostate cancer with rectal wall involvement. Nine elderly patients with such cancer, with a mean age of 74.75 ± 7.19 years, were included. Specimens were obtained using biopsy in 5 patients, fine needle aspiration (FNA) in 3 patients, and surgical resection in 1 patient who underwent 3 months of chemotherapy prior to surgery to shrink the tumor.

### Clinical characteristics and findings

The clinical characteristics and macroscopic findings are summarized in Table 1. The mean patient age was 74 years (range, 64–85 years). Eight patients (64.3%) had no prior history of prostate cancer, whereas 1 had a history of prostate cancer (9 years earlier). Symptoms included a change in bowel movements (*n* = 4), rectal urgency (*n* = 4), pelvic pain (*n* = 1), rectal mass (*n* = 2), and lower gastrointestinal bleeding (*n* = 2). Only 1 patient had prostate-related symptoms including urinary frequency, difficulty in urination, and painful urination.

**Table 1:** Clinical, demographic and macroscopic findings

Case	Type	Age	Level of total serum PSA(ng/ml)	Ration of free PSA to unbound PSA	Symptoms	Gross/Endoscope	Clinical diagnosis	Clinical treatment after pathologic diagnosis	Prognosis (Months)
1	FNAs	66	83.32	Low	Change in bowel movements	Protruded lesion, 3 centimeter away from anus	Rectal carcinoma	Standard treatment based on prostate cancer	8(alive)
2	Biopsy	79	>100	Low	Rectal urgency, lower gastrointestinal bleeding,	Rectal ulcer lesion, 4 centimeter away from anus	Rectal carcinoma	Standard treatment based on prostate cancer	87(alive)
3	Biopsy	85	Unknown	Low	Rectal urgency, change in bowel movements,	Rectal circumferential mass, 5 centimeter away from anus	Rectal carcinoma	Standard treatment based on prostate cancer	69(alive)
4	Biopsy	64	Unknown	Low	Change in bowel movements	Rectal circumferential mass, 3 centimeter away from anus	Prostate cancer involving rectum	Standard treatment based on prostate cancer	68(alive)
5	FNAs	80	83.04	Low	Rectal urgency, Rectal mass	Rectal circumferential mass, 4 centimeter away from anus	Rectal carcinoma	Standard treatment based on prostate cancer	54(alive)
6	FNAs	74	>100	Low	Rectal urgency, Pelvic pain and prostate-related symptoms	Protruded lesion in anterior rectal wall, 2 centimeter away from anus	Gastrointestinal stromal tumor	Standard treatment based on prostate cancer	45(alive)
7	Biopsy	72	55.68	Low	Lower gastrointestinal bleeding	Crater-like tumor mass in rectum, 3 centimeter away from anus	Rectal carcinoma	Standard treatment based on prostate cancer	32(alive)
8	Biopsy	78	91.01	Low	Change in bowel movements	Protruded lesion,7 centimeter away from anus	Rectal carcinoma	Standard treatment based on prostate cancer	19(alive)
9	Resection	67	>100	Low	Rectal urgency	Protruded lesion in rectum, 6 centimeter away from anus	Rectal carcinoma	Surgical operation and then standard treatment based on prostate cancer	6(dead)

Endoscopy revealed that the tumor masses were located at 2–7 cm away from the anus, and ranged 1–6 cm in size. Grossly, the tumors included circumferential rectal masses (*n* = 3), an ulceration lesion (*n* = 1), a crater-like mass (*n* = 1), and protruding lesions (*n* = 4). In 7 patients, serum PSA levels were 6–10 times higher than the upper limit of the normal level; the PSA level was not recorded in 2 patients. The ratio of free PSA to unbound PSA was low in all 9 patients. The primary clinical impression in these cases was rectal carcinoma (*n* = 7), gastrointestinal stromal tumor (*n* = 1), and prostate cancer involving the rectum (*n* = 1).

### Histopathological examination

A summary of the histopathological findings is presented in Table 2. There was no evidence of intraepithelial neoplasia in any case. There were 2 cases of cancer cell invasion in the mucosa, 7 cases of submucosa invasion, and all cases had muscularis propria involvement. The Gleason score system is based on the degree of glandular architecture, differentiation, and the tumor growth pattern, and is by far the best predictor of prostate tumor progression and prognosis. The Gleason score in all patients was ≥7 (Table 2).

**Table 2:** Histopathologic findings

Case	Type	Gleason score	Mucosa involvement	Submucosa involvement	muscularis propria involvement	Intraepithelial neoplasia of the rectal mucosal epithelium	IHC staining	Final Diagnosis
1	FNAs	5 + 4	No	No	Yes	No	P504S(+), PSA(+),CK20(−), CDX2(−)	Prostate cancer involving rectum
2	Biopsy	5 + 5	Yes	Yes	Yes	No	P504S(−), PSA(+),CK20(−), Villin(−)	Prostate cancer involving rectum
3	Biopsy	4 + 4	No	Yes	Yes	No	P504S(+), PSA(+),CK20(−), CDX2(−)	Prostate cancer involving rectum
4	Biopsy	4 + 3	No	No	Yes	No	P504S(−), PSA(+),CK20(−), CDX(−)	Prostate cancer involving rectum
5	FNAs	5 + 5	No	Yes	Yes	No	P504S(+), PSA(+),CK20(−), Villin(−)	Prostate cancer involving rectum
6	FNAs	4 + 3	No	Yes	Yes	No	P504S(+), PSA(+),CK20(−), CDX2(−)	Prostate cancer involving rectum
7	Biopsy	3 + 4	No	Yes	Yes	No	P504S(+), PSA(+),CK20(−), Villin(−)	Prostate cancer involving rectum
8	Biopsy	5 + 5	Yes	Yes	Yes	No	P504S(−), PSA(+),CK20 (+), CDX(−)	Prostate cancer involving rectum
9	Resection	3 + 4	No	Yes	Yes	No	P504S(+), PSA(+),CK20(−), CDX-2(−)	Prostate cancer involving rectum

**Fig. 1:**
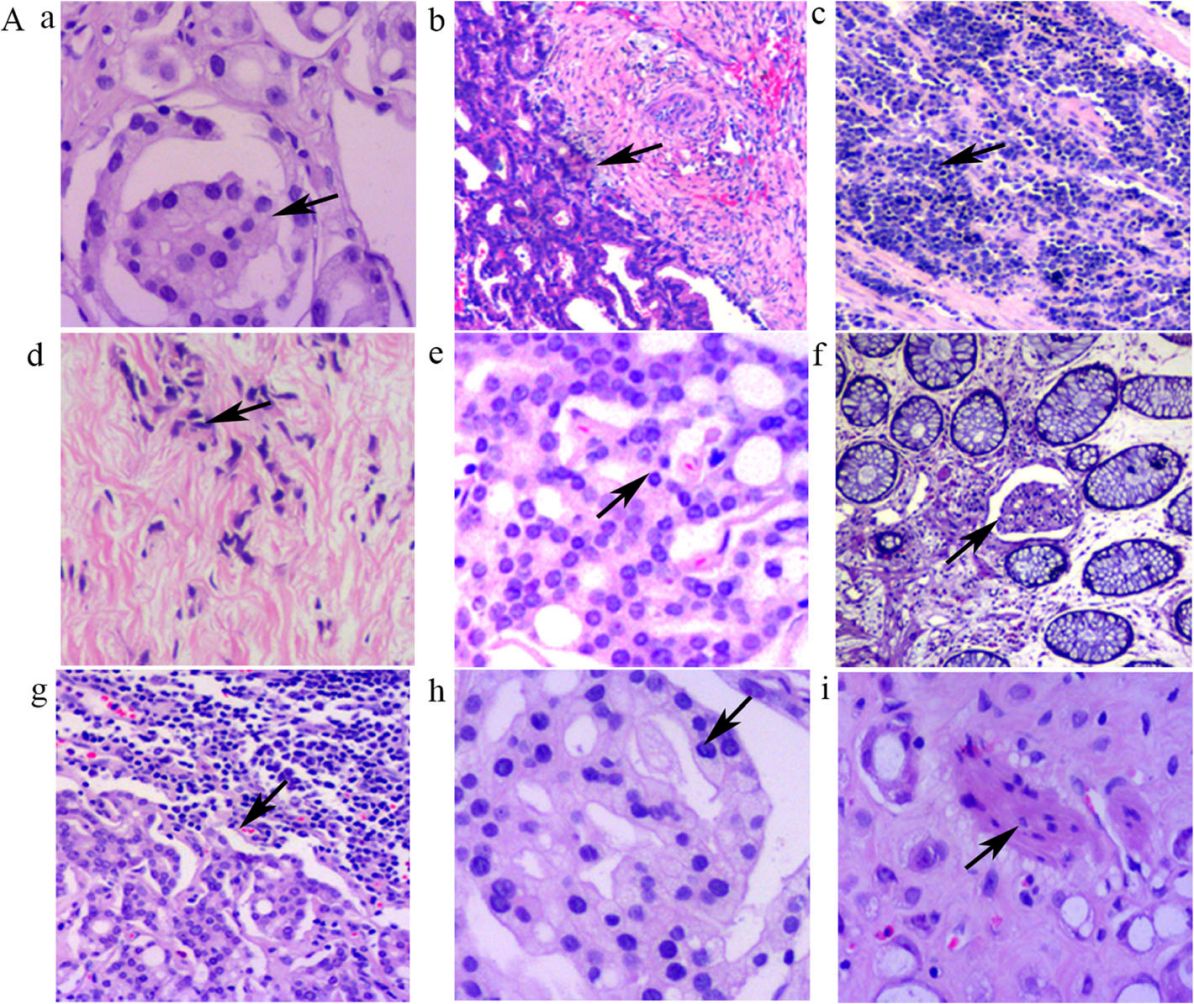
**A**. Morphological characteristics of prostate cancer involving the rectal wall. **a**. Foamy glands (*black arrow heads*). **b**. Small glands (*black arrow heads*). **c**. Diffuse individual cell infiltration (*black arrow heads*). **d**. Small prostate cancer cells infiltrating the muscularis propria (*black arrow heads*). **e** Cribriform structure (*black arrow heads*). **f**. Emboli in the lymph-vessels of the mucosal layer (*black arrow heads*). **g**. Lymph tissue metastasis (*black arrow heads*). **h**. Glomeruloid structures (*black arrow heads*). **i**. Perineural capsular invasion (*black arrow heads*). All images are hematoxylin and eosin (H&E) sections with a magnification of × 200

Microscopically, the tumors exhibited a wide spectrum of appearances including foamy glands caused by accumulation of lipids (Fig. 1A -a), small glands (Fig. 1A -b), diffuse individual cell infiltration (Fig. 1A -c and A -d), and cribriform structures (Fig. 1A -e). In the foamy gland carcinomas, the tumor cells were large, cuboidal, or columnar-shaped, with small and round nuclei and conspicuous nucleoli. The behavior of the foamy gland carcinomas was aggressive with metastases to the lymph tissue (Fig. 1A -g). Furthermore, these architectural patterns were accompanied by cytological abnormalities in the form of nuclear enlargement, inconsistent nucleus size, and prominent nucleoli.

In the 9 patients with prostate cancer, 3 had small-sized glands and 2 had medium-sized glands. Diffuse individual cell infiltration was seen in 4 tumors. Lymph vessel emboli were apparent in 2 patients (Fig. 1A -f), and lymph tissue metastatic foci could be observed in 3 patients (Fig. 1A -g). Glomeruloid structure (Fig.1A -h), a morphologic feature used in the diagnosis of prostate cancer, could be found in the rectal foci derived from the prostate cancers. The presence of prostatic cancer cells within the perineural spaces was seen in 4 patients (Fig.1A -i), a morphological feature for pathologic diagnosis of a malignant tumor.

### Immunohistochemical findings

**Fig. 2:**
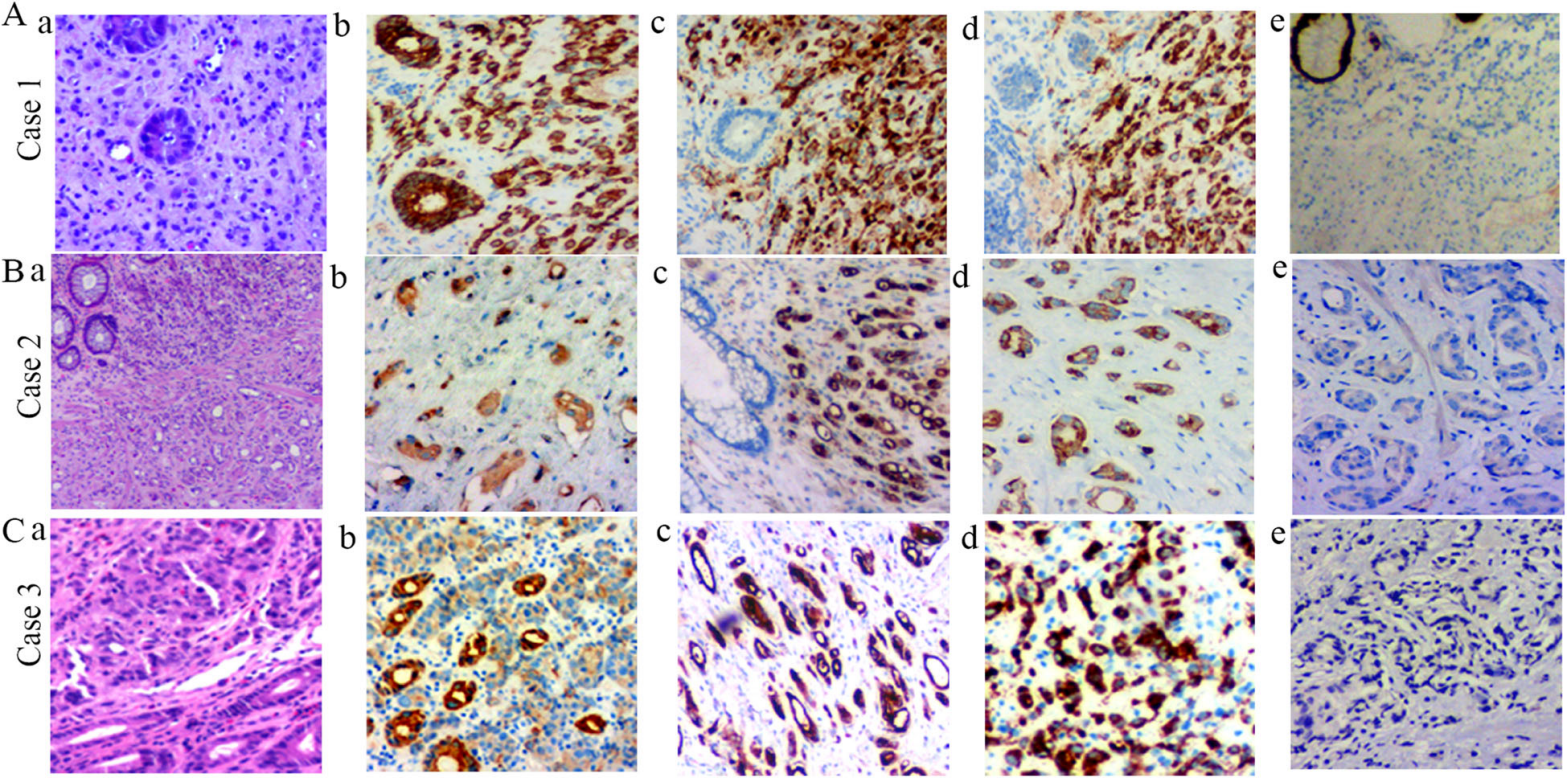
H&E and immunohistochemical (IHC) staining in 3 cases of prostate cancer involving the rectal wall. **A**. IHC staining confirmed the diagnosis of prostate cancer in case 1 (all images, except a, are of IHC staining, magnified × 200). **a**. H&E staining of case 1 (Gleason score 5 + 4). **b**. Strong positive expression of CK in prostate cancer and mucosal epithelial cells. **c** and **d**. The cancer cells are strongly positive for PSA and P504s, whereas the epithelial cells of the rectal mucosa are negative. **e**. The cancer cells are negative for CDX2. **B**. IHC staining confirmed the diagnosis of prostate cancer in case 2 (all images, except **a**, are of IHC staining, magnified × 200, except **b**, magnified × 100). **a**. H&E staining of case 1 (Gleason score 4 + 3). **b**. Positive expression of CK in cancer cells. **c**. Strong positive expression of PSA in cancer cells with no expression in rectal epithelial cells. **d**. Strongly positive expression of P504s in cancer cells. **e**. The cancer cells are negative for villin. **C**. IHC staining confirmed the diagnosis of prostate cancer in case 3 (all images, except **a**, are of IHC staining, magnified × 200). **a**. H&E staining in case 3 (Gleason score 4 + 4). **b**. The cancer cells are weakly positive for CK and the epithelial cells are strongly positive. **c** and **d**. The cancer cells are strongly positive for PSA and P504s. **e**. The cancer cells are negative for CK20

All tumors were PSA and CK-pan positive. Six tumors were P504s-positive. Staining for CK20, villin, and CDX2, which are important indicators of tumors derived from the digestive system, were negative (Fig. 2A -C). Fig. 2 shows the IHC findings in 3 cases of prostate cancer involving the rectum. In these 3 cases, the results of hematoxylin and eosin staining showed atypical cells and glandular architectures distributed among the rectal epithelial cells (Fig. 2A -a, B -a and C -a). These atypical cells were positive for CK-pan IHC staining (Fig. 2A -b, B -b and C -b), which confirms their origin as epithelial. The cancer cells in all 3 cases were strongly positive for PSA (Fig. 2A -c, B –c, and C -c) and P504s (Fig. 2A -d, B -d and C -d). In all 3 cases, the cancer cells were negative for CDX-2, villin, and CK20 (Fig. 2A -e, B -e, C -e).

Follow-up information was available for all patients. As of March 31, 2015, 8 patients were alive at follow-up (mean, 47.75 months; range, 8–87 months). The 7 patients who underwent surgical resection and endocrine therapy based on a diagnosis of prostate cancer were still alive. The patient with a prostate cancer history underwent endocrine therapy and surgical resection and is still alive. One patient who underwent surgical resection and subsequently underwent standard rectal cancer therapy died 6 months posttreatment because of extensive metastases from the prostate cancer.

## Discussion

Prostate cancer most commonly metastasizes to the bones and lymph nodes or directly infiltrates to the bladder and ureters [16]. Digestive tract metastasis from prostate adenocarcinoma is relatively rare. There is a thick layer of fascia (Denonvilliers’ fascia) between the prostate and the rectum, making it difficult for prostate cancer to invade the rectum. There are 3 potential routes for prostate cancer to invade the rectal wall, including prostate cancer directly invading through Denonvilliers’ fascia and infiltration into the rectum, lymphatic metastasis, and prostate cancer cells spreading through needle biopsy to seed into peri-rectal or rectal tissue [7].

Because the morphologic features of prostate cancer and rectal adenocarcinoma are similar, there is a risk of misdiagnosis, which can have adverse consequences for the patient because of the subsequent use of inappropriate treatment strategies [7, 17]. There are fundamental differences in treatment for these two kinds of malignancy. Treatment of aggressive prostate cancers may involve surgery, radiation therapy, chemotherapy, or combination therapy [18–20]. In addition, some elderly patients are not offered curative treatment options and instead made to undergo hormonal therapy or careful observation [21]. Furthermore, hormonal therapy and chemotherapy are often reserved for disease that has spread beyond the prostate. In contrast, for localized rectal cancer, the preferred treatment is complete surgical resection with adequate margins, although chemotherapy is often used preoperatively to shrink the tumor.

Here, we reviewed the data of 9 patients with prostate cancer involving the rectal wall, only 1 of whom had a history of prostate cancer. They were all clinically diagnosed with rectal carcinoma. One patient who underwent chemotherapy for a clinical diagnosis of rectal carcinoma, prior to the correct diagnosis of prostate cancer, died shortly after treatment because of prostate cancer metastasis, which emphasizes the importance of an accurate and timely diagnosis.

Rectal examination is an efficient method for the detection of prostate cancer. However, a pathological confirmation using biopsy or FNA is necessary for a definitive diagnosis of prostate cancer [22, 23] because of the high incidence of both cancers in elderly patients. Differences in pathology between prostate and rectal cancers, including cytoarchitecture, glandular architecture, cellular pleomorphism, and mucosecretion patterns, can facilitate a correct diagnosis [24]. In rectal adenocarcinoma, tumor cells form irregular tubular structures, have multiple lumens, and have reduced stroma [25]. In contrast, prostate cancer glands have irregular outlines with a smooth inner surface. In addition, the epithelial cells in the rectal mucosa often exhibit intraepithelial neoplasia [26]. In the present study, there was no evidence of intraepithelial neoplasia in the rectal mucosa layer, suggesting that the tumors may be not rectal carcinomas. Furthermore, the tumor cells in the present study exhibited the 4 major cytoarchitectural patterns associated with prostate cancer including medium-sized glands, small glands, diffuse individual cell infiltration, and cribriform patterns. The prostate cancer cells present with nuclear enlargement and prominent nucleoli [26, 27].

IHC to identify specific markers of prostate and colorectal cancers plays an important role in confirming the origin of the tumor. Liu et al. reported that IHC staining is an essential tool in distinguishing the origin of metastatic cancer, particularly in cases where the histology does not appear typical of rectal carcinoma [15]. PSA and P504s are both specific, accurate, commonly used markers of prostate cancer cells. PSA is secreted specifically by the epithelial cells of the prostate gland [28], and PSA serum levels are often elevated in patients with prostate cancer [29, 30]. In the blood, the majority of PSA is bound to serum proteins with a small fraction of unbound PSA. Therefore, in patients with prostate cancer, the ratio of free PSA to unbound PSA is significantly decreased, providing a powerful diagnostic tool [31]. P504s is a highly sensitive marker for prostate cancer that is useful for the detection of small foci in biopsy and FNA specimens [32, 33]. However, P504s is not always present in prostate cancer because the degree of tumor differentiation can affect its expression. Furthermore, in contrast to rectal cancer, prostate cancer is rarely positive for CK20 [34–36]. Villin is localized to the microvilli of the brush border of the intestinal epithelium and is a good marker of adenocarcinomas of intestinal origin. CDX2 is a highly sensitive and specific marker of adenocarcinomas of intestinal origin [37].

In the present study, all tumors were positive for PSA, and 6 tumors were positive for P504s. In contrast, all tumors were negative for CK20, CDX2, and villin [38–40]. In addition, all these tumors were negative for CDX-2; however, some androgen-independent prostate tumors display nuclear CDX2 staining [41], which could represent a potential dangerous pitfall for the differential diagnosis from rectal carcinoma.

## Conclusions

In the present study, we identified a number of considerations to keep in mind when making a differential diagnosis of prostate cancer. First, most colorectal cancers arise from adenomatous polyps [42, 43] and transitional lesions (low-grade or high-grade intraepithelial neoplasia) between the normal epithelium and carcinoma, and often appear in the rectal mucosal layer, which can help judge the origination of rectal lesions. Intraepithelial neoplasia does not occur in rectal metastatic tumors derived from prostate cancer. Second, the medical history of the patient should be carefully evaluated because 1 of the patients in this study presented with a history of prostate cancer. When a tumor mass appears in the rectal wall of patients with a medical history of prostate cancer, the diagnosis of rectal cancer should only be reached after prostate cancer has been ruled out. Third, IHC staining for tissue-specific markers provides significant support, enabling pathologists to determine an accurate and definitive diagnosis. Furthermore, in elderly male patients who have a tumor mass in the rectum, the differential diagnosis of prostate cancer must be performed to avoid misdiagnosis. Any treatment should be postponed until the final definitive diagnosis is reached.

## Abbreviations

H&E: Hematoxylin-eosin
IHC: Immunohistochemical
CK: Cytokeratin
